# The orobasal organ (of Ackerknecht) is present in prenatal mice

**DOI:** 10.3389/fcell.2025.1590311

**Published:** 2025-05-20

**Authors:** Sven Schumann, Jan R. Munk, Michael J. Schmeisser, Moritz Staeber

**Affiliations:** ^1^ Institute of Anatomy, University Medical Center of the Johannes Gutenberg University Mainz, Mainz, Germany; ^2^ Institute of Anatomy, Brandenburg Medical School Theodor Fontane, Neuruppin, Germany; ^3^ Faculty of Health Sciences, Joint Faculty of the Brandenburg University of Technology Cottbus-Senftenberg, The Brandenburg Medical School Theodor Fontane and the University of Potsdam, Brandenburg, Germany; ^4^ Focus Program Translational Neurosciences, University Medical Center of the Johannes Gutenberg-University, Mainz, Germany

**Keywords:** animal model, experimental dentistry, gingiva, oral cavity, oral mucosa, vestigial organs

## Abstract

**Background:**

In 1912, the veterinary anatomist Eberhard Ackerknecht described morphologically highly variable epithelial invaginations behind the medial mandibular incisors. This orobasal organ (of Ackerknecht) is present in different mammalian species including humans, but its presence in mice was under debate in literature. While the function of the orobasal organ is still unknown, it might play a role in the development of cysts of the oral floor.

**Methods:**

H&E-stained histological serial slides of the developing oral floor of C57BL/6J mice embryos were investigated (n = 40).

**Results:**

The orobasal organ was present in mice and developed between prenatal days E15 and E17 (prevalence in E15 embryos: 0%, prevalence in E17 embryos: 90.5%). The organ was present both in male and female embryos. In E17, the organ had an average size of 68.75 (±41.1) μm x 58.75 (±8.5) μm x 345 (±28.3) μm (length x depth x width).

**Discussion:**

While the existence of an orobasal organ was already shown for pre- and postnatal rats, there was only one publication dealing with the orobasal organ in mice. In this study, adult mice were investigated and no orobasal organ was found. Here, we demonstrate the existence of an orobasal organ in mice, at least in embryos. The presence of the orobasal organ in a common model organism will help to investigate its pre- and postnatal development, as well as possible physiological functions of this structure.

## 1 Introduction

Detailed knowledge of oral mucosa anatomy is of the utmost importance in both human and veterinary dentistry. The oral mucosa consists of two layers, the stratified squamous epithelium and the lamina propria. It can be divided into three main types: 1) The lining mucosa, which (in humans) is nonkeratinized, 2) the masticatory mucosa on tongue (dorsum linguae), hard palate, and attached gingiva, which is keratinized, and 3) the specialized mucosa of taste buds (Orban and Sicher, 1945). In 1912, the veterinary anatomist Eberhard Ackerknecht (1883–1968) described morphologically highly variable epithelial invaginations at the mucogingival junction behind the medial mandibular incisors (Ackerknecht, 1912). The orobasal organ (of Ackerknecht) seems to be present in all subclasses of mammals (protheria, marsupialia, eutheria) (Staeber et al., 2023) with the exception of some orders with a highly modified stomatognathic system (e.g., pangolins, whales) (Staeber et al., 2023; García de Los Ríos et al., 2021). While the orobasal organ seems to be present in rats (Ackermann, 1924; Schückher, 1937; Nishiyama, 1933), there is only one publication which deals with its presence in mice. Interestingly, Nishiyama did not find an orobasal organ in the Japanese dancing mouse (Nishiyama, 1933).

Rodents are mammals of the order Rodentia (from the Latin term rodere, to gnaw), which are characterized by a single pair of continuously growing incisors in each of the upper and lower jaws. According to Westheide and Rieger, they can be classified into five suborders: 1) Anomaluromorpha, 2) Castorimorpha, 3) Hystricomorpha, 4) Myomorpha and 5) Sciuromorpha (Westheide and Rieger, 2015). With mice, rats, hamsters, and gerbils, the suborder Myomorpha contains important pets as well as laboratory animals. The laboratory mouse is a hybrid of different mouse subspecies: *Mus musculus musculus, domesticus, castaneus* and *molossinus*. With 68%–92%, the laboratory mouse genome mostly derives from *Mus musculus domesticus* (Linder and Davisson, 2012). The laboratory mouse is the most commonly used mammalian research model and is widely used in experimental dentistry (Nokhbatolfoghahaei et al., 2020). The oral mucosa of the laboratory mouse is characterized by an orthokeratinized squamous epithelium. The thickness of the stratum corneum varies with diet and frequency of food uptake (Treuting et al., 2018).

The function of the orobasal organ is completely unknown, so far. However, defects in its development might result in the formation of cysts of the oral floor (Ungerecht, 1951). In order to find a potential model organism to analyze the function of the orobasal organ, this study aimed to identify its presence in the laboratory mouse.

## 2 Materials and methods

All experiments were carried out in accordance with German laws for animal protection. Ethical review and approval were not required for the animal study because organ removal from mice for scientific purpose does not require approval by an ethics committee in Germany. Pregnant mice (C57BL/6J) (n = 8) were sacrificed by cervical dislocation and the uterus was removed. Embryos on embryonic days E15 (n = 19) and E17 (n = 21) were sacrificed by decapitation. The tip of the tail was removed for sex identification by PCR. The PCR was performed with the following primers: SX_R, 5′-GATGATTTGAGTGGAAATGTGAGGTA-3′ and SX_F, 5′-CTTATGTTTATAGGCATGCACCATGTA-3′. A standard PCR protocol was used as follows: 94°C for 2 min followed by 35 cycles of 94°C for 30 s, 57°C for 30 s, 55°C for 30 s, 72°C for 30 s and 72°C for 5 min. PCR showed amplicons of 280 bp for males and 685 bp for females.

To exclude effects of tissue fixation on mucosal morphology (e.g., shrinkage of the tissue leading to artificial epithelial folds), embryonic heads were fixated either in 4% buffered formaldehyde solution or with Bouin´s solution [aqueous solution of picric acid (0.9%), acetic acid (5%), and formaldehyde (9%)].

For histological evaluation, hematoxylin and eosin (H&E) staining was performed after paraffin embedding of the samples. Five-micrometer thick serial sections of the embryonic heads were prepared. Sections were deparaffinized in xylene and rehydrated in ethanol with decreasing concentration. H&E staining was performed with hematoxylin (Sigma-Aldrich, St. Louis, Missouri, United States) and eosin (Sigma-Aldrich) after rinsing with distilled water. Staining was followed by dehydration in ethanol with increasing concentrations, and treatment in xylene. Histological sections were photographed using a Leica MS 5 tripod (Leica Microsystems, Germany) and a JVC KY-F75U C-mount digital camera (JVC, Yokohama, Japan). Measurements were performed in Fiji software for length (x-axis) and depth (y-axis). The width of the orobasal organ (z-axis) was reconstructed based on the thickness of the serial sections. The first and last serial section with a visible orobasal organ was determined. The number of sections with a visible orobasal organ was multiplied with 5 μm. All calculations were made with the Microsoft Excel software.

## 3 Results

**FIGURE 1:**
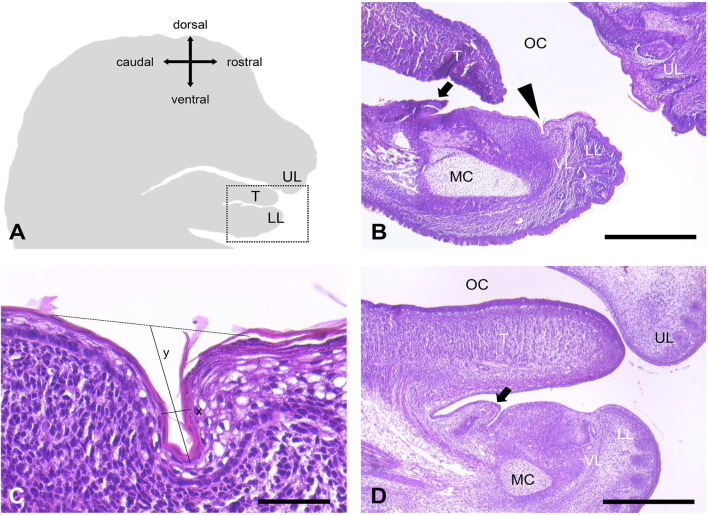
Orobasal organ in mice. **(A)** Schematic representation of a sagittal section of a mouse head. The snout points to the right. The region shown in B is marked with a dotted square. **(B)** The orobasal organ (arrowhead) was localized between the vestibular lamina (VL) and the sublingual caruncle (arrow) in animal no. 21 (E17, female). Oral cavity (OC), upper lip (UL), lower lip (LL), tongue (T) and Meckel´s cartilage (MC) were marked. **(C)** Orobasal organ in higher magnification. Measured distances for length (x) and depth (y) were indicated. **(D)** No orobasal organ was visible in animal no. 19 (E15, female). Oral cavity (OC), upper lip (UL), lower lip (LL), tongue (T), Meckel´s cartilage (MC), vestibular lamina (VL) and the sublingual caruncle (arrow) were marked. Measuring bar in **(B, D)** 500 μm, measuring bar in **(C)** 50 μm.

We were able to visualize an orobasal organ in 19 of 21 mice embryos on day 17 of prenatal development (E17) (90.5%). It was localized between the vestibule lamina and the sublingual caruncle (Figures 1A–C). The orobasal organ was found in both male (n = 13) and female embryos (n = 6) (Table 1). One male fixed in Bouin´s solution and one female fixed in formaldehyde showed no signs of an orobasal organ. In no case investigated (n = 19) an orobasal organ was found in an embryo day E15 (0%) (Figure 1D). While general tissue preservation appeared to be superior after fixation with Bouin´s solution in comparison to buffered formaldehyde solution, we observed no obvious influence of tissue fixation (Bouin´s solution vs buffered formaldehyde) on the morphology of the orobasal organ.

**TABLE 1:** Investigated mouse embryos. The presence of an orobasal organ was confirmed histologically.

Developmental age	Number of embryos, n =	Male embryos, n =	Female embryos, n =	Male embryos with an orobasal organ, n =	Female embryos with an orobasal organ, n =	Embryos fixated with buffered formaldehyde solution, n =	Embryos fixated with Bouin´s solution, n =
E15	19	6	13	0	0	10	9
E17	21	14	7	13	6	11	10

**FIGURE 2:**
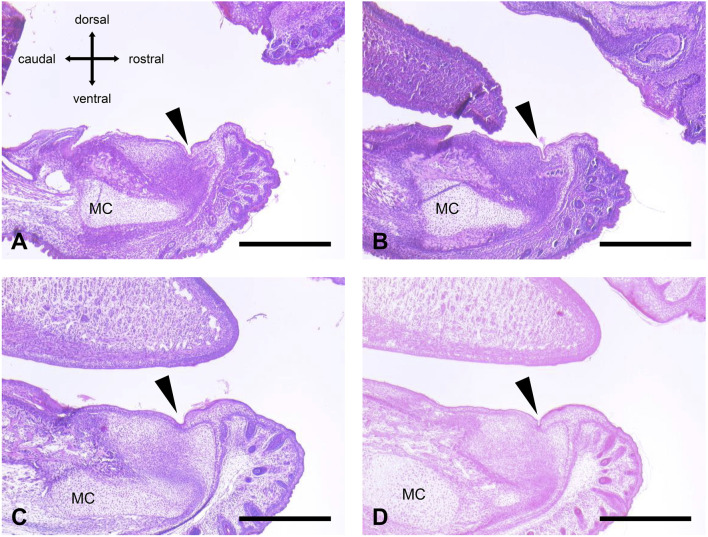
Orobasal organ in four different E 17 embryos. **(A)** Animal no. 20 (male, fixation with formaldehyde solution). **(B)** Animal no. 21 (female, fixation with formaldehyde solution). **(C)** Animal no. 32 (female, fixation with Bouin´s solution). **(D)** Animal no. 40 (male, fixation with Bouin´s solution). The orobasal organ (arrowhead) and Meckel´s cartilage (MC) were marked. See also Table 2. Measuring bar in **(A–D)**: 500 μm.

We performed measurements of four representative orobasal organs (Figures 1C, 2; Table 2). In E17, the orobasal organ had an average size of 68.75 (±41.1) μm x 58.75 (±8.5) μm x 345 (±28.3) μm (length x depth x width) (n = 4).

**TABLE 2:** Histomorphometric analysis of four different representative orobasal organs in E17 embryos.

Animal no.	Sex	Fixative	Length in µm	Depth in µm	Estimated width in µm
20	Male	Buffered formaldehyde	55	55	345
21	Female	Buffered formaldehyde	20	70	385
32	Female	Bouin´s solution	80	50	325
40	Male	Bouin´s solution	120	60	325
Average			**68.75**	**58.75**	**345**
Standard deviation			42.1	8.5	28.3

Average (bold number) is the mean value of the four animals.

## 4 Discussion

**FIGURE 3:**
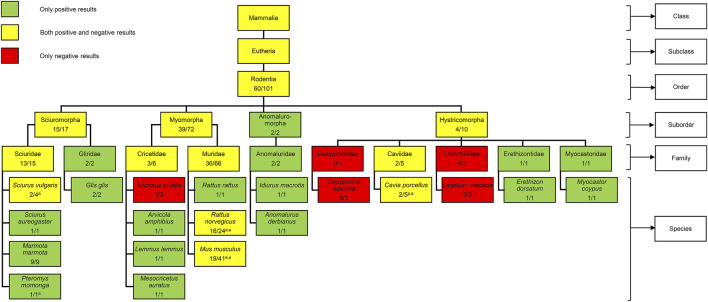
Presence of the orobasal organ in different rodents. No member of the suborder Castorimorpha was investigated. The number before the slash indicates the minimum number of positive findings while the number behind the slash indicates the minimum number of investigated individuals. Embryos (n = 40) and positive findings (n = 19) of the present study were included. The age of the animals investigated is indicated with a superscript letter (e = embryo; p = postnatal; a = adult). In several cases, the age of the animals investigated is unknown. For information about mammals in general, see Staeber et al., 2023.

The orobasal organ has been the subject of research for more than 100 years and was detected in many different mammalian species, including humans (Schückher, 1937; Kato, 1953; De Risky, 1954; Zorzoli, 1954; Kagawa, 1956; Staeber and Schumann, 2022). In rodents, the orobasal organ was already investigated in five studies: Keller (Keller, 1921), Ackermann (Ackermann, 1924), Nishiyama (Nishiyama, 1933), Schückher (Schückher, 1937) and Künzel (Künzel, 1953). An orobasal organ was found in members of the suborders Sciuromorpha, Myomorpha, Anomaluromorpha and Hystricomorpha (Figure 3). Unfortunately, no member of the suborder Castorimorpha was investigated, so far. In the family Muridae, an orobasal organ was found in *Rattus rattus* (Ackermann, 1924) and *Rattus norvegicus* (Nishiyama, 1933; Schückher, 1937). However, for mice, only an unknown number of adult Japanese dancing mice was investigated, so far. Interestingly, no orobasal organ was found in these animals (Nishiyama, 1933). The Japanese dancing mouse or waltzing mouse derived from the Japanese house mouse before 1800 as a mutation with a characteristic black and white fur colour, small body size and movement disorders. The dancing or waltzing is caused by vertigo due to abnormalities of the inner ear. Japanese house mice are considered to belong to either *Mus musculus musculus* or *Mus musculus castaneus*, or to hybrids of these two subspecies (Cruz et al., 2024). Unfortunately, no further information about the origin of the mice was given by Nishiyama. It remains unclear whether the absence of the orobasal organ in these animals is a result of postnatal regression or developmental agenesis.

Taken together, a minimal number of 101 rodents was investigated and the orobasal organ was found in at least 60 cases, so far. Unfortunately, the exact age of the investigated rodents is not mentioned in the older publications. The rats investigated by Schückher were embryos, Keller investigated postnatal guinea pigs and Nishiyama analysed adult specimens of *Sciurus vulgaris*, *Pteromys momonga*, *Rattus norvegicus, Cavia porcellus* and *Mus musculus*. Since our results show that the presence of an orobasal organ depends on the developmental age, future research on this structure must take this factor into account.

In his investigations on *Rattus norvegicus* Schückher was able to determine the sex of his specimens in 14 cases (8 males, 6 females). Interestingly, an orobasal organ was only found in female embryos (n = 4) (Schückher, 1937). In contrast, we were able to find an orobasal organ in both male and female mice.

There are several theories about the origin and function of the orobasal organ. Keller hypothesized, that the orobasal organ is a remnant of an anterior sublingual gland found in reptiles (Keller, 1921). However, no experimental evidence is supporting this theory. Histologically, the orobasal organ does not show any signs of secretory activity or innervation (Malinovsky et al., 1996). Clinically, it was assumed that the orobasal organ might serve as an origin of dermoid cysts of the oral floor (Ungerecht, 1951). Additionally, it could be confused with precancerous oral lesions.

Concerning tissue fixation, our results are in accordance with findings by Mirzaei et al. (2023) who showed that for tissues that are soft and fragile, Bouin´s fixative is more suitable. Unfortunately, tissue fixation with Bouin´s solution could lead to problems when performing consecutive immunohistochemistry (Lenz et al., 2022).

However, there are important limitations in this study. First, the main goal of this study was to confirm the presence of the orobasal organ in the laboratory mouse. Further research must clarify the cellular mechanisms of its development (epithelial proliferation, invagination) and the underlying genetics. Furthermore, our investigations focussed on prenatal developmental staged. This was to avoid the process of decalcification prior to histological analysis. Decalcification can lead to tissue damage and increase artefacts (Taqi et al., 2018). Further research must show if this structure degenerates during lifetime.

Taken together, out study demonstrates the presence of an orobasal organ in *Mus musculus* for the first time. Further studies will have to show whether the orobasal organ is preserved in adult mice or whether it degenerates during their lifetime. Further investigations must clarify the exact developmental mechanics and the fine structure of this organ and can serve as a basis for functional studies on this interesting structure.

## Data Availability

The original contributions presented in the study are included in the article/supplementary material, further inquiries can be directed to the corresponding author.

## References

[B1] Ackerknecht E. (1912). Ein eigenartiges Organ im Mundhöhlenboden der Säugetiere. Anat. Anz. 41, 434–449.

[B2] Ackermann O. (1924). Neues über das Vorkommen des Ackerknecht’schen Organs in der Säugetierreihe. Anat. Anz. 57, 449–473.

[B3] Cruz M. Bergmans W. Takada T. Shiroishi T. Yoshiki A. (2024). Type specimens, taxonomic history, and genetic analysis of the Japanese dancing mouse or waltzer, *Mus wagneri* variety *rotans* Droogleever Fortuyn, 1912 (Mammalia, Muridae). ZooKeys 1200, 27–39. 10.3897/zookeys.1200.118823 38736700 PMC11082488

[B4] De Risky S. (1954). The organ of Ackerknecht in humans. Rassegna Odontotec. 35 (1), 61–67. 13177981

[B5] García de Los Ríos Á. Soler Laguía M. Arencibia Espinosa A. Martínez Gomariz F. Sánchez Collado C. López Fernández A. (2021). Endoscopic study of the oral and pharyngeal cavities in the common dolphin, striped dolphin, risso's dolphin, harbour porpoise and pilot whale: reinforced with other diagnostic and anatomic techniques. Anim. (Basel) 11 (6), 1507. PMID: 34067447; PMCID: PMC8224762. 10.3390/ani11061507 PMC8224762 34067447

[B6] Kagawa G. (1956). Über das früheste Stadium der Entwicklung der Zähne des Menschen. Arch. Histol. Jpn. 10, 483–523. 10.1679/aohc1950.10.483

[B7] Kato K. (1953). Embryological studies on the development of the tooth in human embryo, with special reference to the formation of the tooth band and the lip furrow band. Shigaku 41, 3–54.

[B8] Keller E. (1921). Über ein rudimentäres Epithelialorgan im präfrenularen Mundboden der Säugetiere. Anat. Anz. 55, 265–285.

[B9] Künzel E. (1953). Das ackerknecht’sche organ beim syrischen goldhamster. Berl. Münchener Tierärztliche Wochenschr. 11, 175–176.

[B10] Lenz J. Macháčová D. Konecná P. Fiala L. Kyllar M. Tichý F. (2022). Effects of different fixatives over different fixation times, including Antigenfix, on immunohistochemical studies. Acta Veterinaria Brno 91, 179–188. 10.2754/avb202291020179

[B11] Linder C. Davisson M. (2012). “Historical foundations,” in Hedrich (hrsg.), *the laboratory mouse* . Second Edition (Elsevier), 21–35. 10.1016/B978-0-12-382008-2.00002-7

[B12] Malinovsky L. Umlauf R. Cavallotti C. Malinovska V. Hees H. D'andrea V. (1996). Sensory systems around cavum oris in man. Dtsch. Z. für Akupunkt. 39, 136–139.

[B13] Mirzaei M. Eshaghi-Gorji R. Fani F. Karimpour Malekshah A. Talebpour Amiri F. (2023). Comparative evaluation of the effect of three types of fixatives (formalin, Bouin and Carnoy) on histomorphological features of various viscera. Anat. Histol. Embryol. 52 (6), 882–889. Epub 2023 Jul 1. PMID: 37392057. 10.1111/ahe.12945 37392057

[B14] Nishiyama Y. (1933). Beiträge zur Kenntnis der Morphologie und Entwicklung des Ackerknecht’schen Organs. Keizyo J. Med. 4, 417–433.

[B15] Nokhbatolfoghahaei H. Paknejad Z. Bohlouli M. Rezai Rad M. Khojasteh A. (2020). “Animal models in dental research,” in Applications of biomedical engineering in dentistry. Editor Tayebi L. (Cham: Springer). 10.1007/978-3-030-21583-5_18

[B16] Orban B. Sicher H. (1945). The oral mucosa. J. Dent. Educ. 10, 94. 20984632

[B17] Schückher R. (1937). Embryologische Untersuchungen über das Ackerknechtsche Organ bei Ratte und Mensch. Z. für mikroskopisch-anatomische Forsch. 41, 558–568.

[B18] Staeber M. Schumann S. (2022). The orobasal organ of Ackerknecht in a male body donor: a case report. Eur. J. Anat. 26, 237–239. 10.52083/PWMD5478

[B19] Staeber M. Storsberg S. D. Schumann S. (2023). The orobasal organ (of Ackerknecht): a bizarre structure of the mammalian oral cavity. J. Morphol. 284, e21589. 10.1002/jmor.21589 37183493

[B20] Taqi S. A. Sami S. A. Sami L. B. Zaki S. A. (2018). A review of artifacts in histopathology. J. Oral Maxillofac. Pathol. 22 (2), 279. PMID: 30158787; PMCID: PMC6097380. 10.4103/jomfp.JOMFP_125_15 PMC6097380 30158787

[B21] Treuting P. M. Morton T. H. Vogel P. (2018). “7 - oral cavity and teeth,” in Comparative anatomy and histology. Editors Treuting P. M. Dintzis S. M. Montine K. S., Second Edition (Academic Press), 115–133. 10.1016/B978-0-12-802900-8.00007-5

[B22] Ungerecht K. (1951). Zur Genese der Dermoidcysten am Mundboden. Archiv für Ohren. Nasen‐ Kehlkopfheilkd. 160 (4), 316–327. 10.1007/bf02112382

[B23] Westheide W. Rieger G. (2015). “Spezielle zoologie,” in Teil 2: Wirbel-und Schädeltiere. Berlin: Springer Spektrum.

[B24] Zorzoli E. (1954). Ackerknecht's organ in animals and man. Biol. Lat. 7 (3), 585–597. 13199019

